# Supplementary material for: A Personalized Approach to Maintaining Brain Drainage: A Case Series with a Technical Note
Source: J Pers Med. 2025 Jun 20;15(7):264. doi: 10.3390/jpm15070264 (PMC12300991; doi:10.3390/jpm15070264)
Supplement: Supplementary file 1 [file jpm-15-00264-s001.zip › jpm-3598410-supplementary.pdf]

Supplementary material: Procedure for the administration of uPA in the EVD

Preparatory phase:

- Close the 3-way valve of the EVD circuit towards the downstream/dripping chamber.

- Open a pack of sterile gauze, wet them with 2% chlorhexidine and wrap the tap from which the sample will be taken, leave the disinfectant to act for 15 minutes

- The second operator lifts the EVD system, without direct contact but using disinfected gauze. (Figure S1a). The first operator places the sterile sheet under the tap system which the second operator will drop onto the sterile field, retaining the disinfected gauze.

- Through the use of sterile adhesive sheets, a field is created which leaves isolated, as far as possible, only the tap system from which the sampling will be carried out.

Operational phase

- 2 ml of lactated ringer are sterilely aspirated and injected into the urokinase vial containing 100,000 IU to recompose the solution.

- The operator opens the tap system, connects an empty 5 ml syringe and withdraws not less than 2 ml of CSF (Figure S1b). This sampling is carried out by keeping the plunger of the syringe under slight tension without carrying out a frank aspiration, the CSF pressure allows the syringe to be filled. Sometimes, in case of subocclusion of the intracranial catheter, the result is partial. [33]

- The operator connects the uPA syringe, previously prepared, containing 1 ml (50,000 IU). Any air bubbles created in the tap during the disconnection and connection phases must be promptly sucked out with the help of a syringe pointing downwards.

- Injection of the solution and disconnection of the syringe.

- Connection of the second syringe containing the 2 ml of pure lactated Ringer's and infusion of the above to push the drug inside the circuit towards the proximal end. If the volume of CSF withdrawn is less than expected, the irrigation volume with lactated ringer will be proportionately reduced.

- Closing the tap and replacing it with new sterile caps.

The EVD circuit remains closed for at least 30 minutes, keeping the ICP values reading, after resetting them to zero (Figure S1c). Any sustained increases in ICP must be promptly reported and treated with possible early reopening of the drainage [34]. After the waiting period, the drainage is reopened and maintained at the level of the external acoustic meatus.

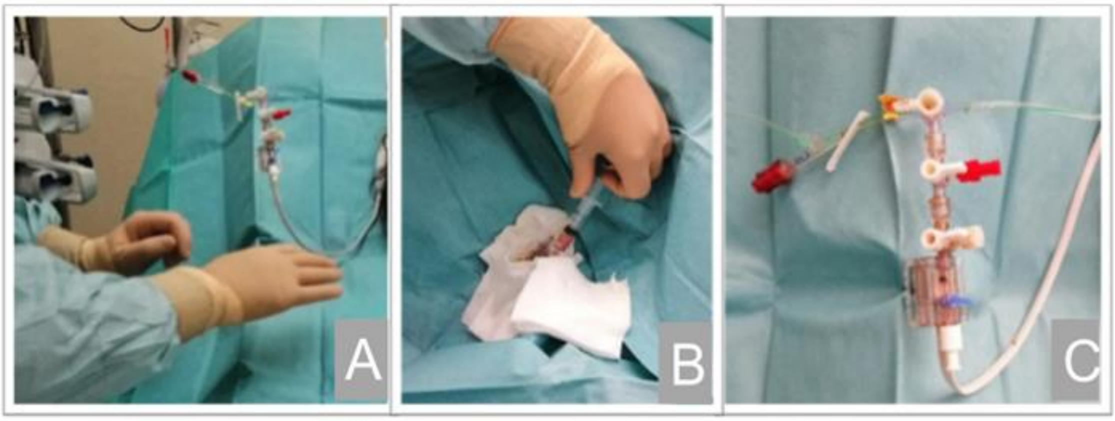

Figure S1. **Procedure for the administration of uPA in the EVD. Figure S1 a-b-c: A. positioning of the sterile drape under the non-sterile drainage B. de-liquoring C. closing of the drain during ICP reading**
